# The relationship between the severity of pulmonary fibrosis and the lung cancer stage

**DOI:** 10.7150/jca.51445

**Published:** 2021-03-14

**Authors:** Hye Jin Jang, Moo Suk Park, Young Sam Kim, Joon Chang, Jae Ho Lee, Choon-Taek Lee, Sang Hoon Lee, Ho Il Yoon

**Affiliations:** 1 Division of Pulmonology, Department of Internal Medicine, Institute of Chest Diseases, Severance Hospital, Yonsei University College of Medicine, 50-1 Yonsei-ro, Seodaemun-gu, Seoul 03722, Republic of Korea.; 2 Division of Pulmonary and Critical Care Medicine, Department of Internal Medicine, Seoul National University Bundang Hospital, 82 Gumi-ro, 173 Beon-gil, Bundang-gu, Seongnam-si, Gyeonggi-do, 463-707, Republic of Korea.

**Keywords:** idiopathic pulmonary fibrosis, lung cancer, GAP stage

## Abstract

**Background:** The incidence of idiopathic pulmonary fibrosis (IPF) and mortality related to the disease have steadily increased in recent years. The risk of cancer is approximately eight times higher in IPF patients than in the general population. The purpose of this study is to determine whether the severity of IPF is related to the time interval between IPF diagnosis and lung cancer diagnosis and to the stage of lung cancer at diagnosis.

**Methods:** In this retrospective cohort study, we reviewed the medical records of patients with lung cancer after IPF diagnosis from two tertiary hospitals in South Korea between 2003 and 2018. We identified 61 patients diagnosed with lung cancer at least 3 months after being diagnosed with IPF.

**Results:** The included patients had a mean age of 71.0 years, and all but one were men (98.4%). The interval between IPF diagnosis and lung cancer diagnosis was not related to the gender-age-physiology (GAP) stage (p=0.662). However, in cox proportional hazard models, a higher GAP stage was significantly correlated with an advanced lung cancer stage (odds ratio 11.1, p=0.003).

**Conclusions:** The lung cancer stage at diagnosis was higher in patients with a higher GAP stage than in those with a lower GAP stage. Physicians should consider implementing more frequent surveillance with computed tomography scans for patients with advanced IPF.

## Introduction

The incidence of idiopathic pulmonary fibrosis (IPF), a debilitating fibrotic lung disease of unknown origin, is steadily increasing, as is the mortality associated with the condition 1. The prognosis of patients with IPF is poor, and there is no curative treatment. The median survival time following the diagnosis is approximately 3 years 2. Although the causes of IPF remain unknown, some risk factors have been identified, including advanced age; smoking; and inhalation of stone, metal, wood, and organic dust 3. IPF and lung cancer (LC) share risk factors (e.g., smoking and advanced age), and pulmonary fibrosis itself is a risk factor for LC 4-7. The relative risk of IPF patients developing LC is approximately eight times higher than that of the general population 8, and the reported prevalence of LC in patients with IPF ranges from 2.7% to 31.3% 6, 9. The incidence of LC also increases with advancement in the clinical course of IPF; the cumulative incidence at 10 years of follow-up exceeds 50% 9-11.

Pulmonary fibrosis and LC also share multiple genetic, molecular, and cellular processes that predispose patients to the diseases 4, 12. Although the precise properties of fibrosis that lead to the development of carcinoma are unknown, one possibility is that progressive scarring causes lymphatic obstruction, which results in a local increase of potentially carcinogenic material 7. Takahashi *et al*. 13 reported high concentrations of carcinoembryonic antigen (CEA) in the bronchoalveolar lavage fluid of patients with fibrosing alveolitis, particularly in patients with associated LC. These high CEA levels may be a marker of premalignant metaplasia and hyperplasia and may predict a greater risk of pulmonary carcinoma during the clinical course of pulmonary fibrosis 7. This is consistent with the observed distribution of the severity of fibrotic lesions in patients with IPF, implicating that the inflammatory procedure and bronchiolar squamous metaplasia may contribute to the pathogenesis of cancer 7. Considering their high incidence, mortality rates, and shared pathogenesis and risk factors, clarification of the relationship between IPF and LC and their clinical features is essential for establishing diagnostic and therapeutic strategies.

Previous studies have highlighted the clinical risk factors associated with LC development in IPF patients and examined the clinical characteristics and survival of patients with both conditions (IPF-LC). There is abundant research on the epidemiological and mechanistic links between IPF and LC 14, and many researchers have focused on the incidence, location, or histologic type of the cancer 9, 15, 16. However, the above-mentioned relationships make it likely that the more severe the IPF, the earlier the cancer will develop or progress to an advanced stage. IPF-LC patients are difficult to treat and have poorer clinical outcomes than patients with IPF or LC alone. Therefore, it is important to identify IPF-LC patients in early stages of the process and provide prompt management. However, little is known about the diagnostic and therapeutic management of these patients. Even the most recent American Thoracic Society/European Respiratory Society/Japanese Respiratory Society/Latin American Thoracic Society (ATS/ERS/JRS/ALAT) guidelines, updated in 2018, do not address this crucial issue 17. In this study, we investigated whether the severity of IPF is related to the time interval between IPF diagnosis and LC diagnosis and to the stage of the cancer.

## Materials and Methods

**Figure 1:**
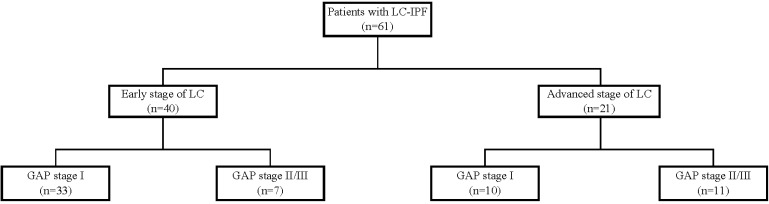
Patient recruitment flow chart.

In this study, we reviewed medical records from two tertiary hospitals in South Korea. Eligible patients had been diagnosed with IPF between 2003 and 2018 and diagnosed with LC at least 3 months later. Figure 1 shows the data collection process. A total of 61 eligible IPF patients were identified. Of these patients, 40 had early stage LC (stages I to IIIA, limited stage of small cell LC) and 21 had advanced stage LC (stage IIIB and higher, extensive stage of small cell cancer). Patients' gender-age-physiology (GAP) index scores were classified into stages I-III, with higher scores indicating greater severity 18. Based on the gender, age, predicted forced vital capacity (FVC), and diffusing capacity of the lung for carbon monoxide (DLco), the GAP index assesses mortality risk levels. Due to the small number of patients in the GAP stage III group (n=5), this group was combined with the GAP stage II group (n=13).

Clinical and laboratory data were collected from patients' medical records. Data on age, sex, body mass index (BMI), smoking history, pulmonary function test results, underlying diseases, Eastern Cooperative Oncology Group (ECOG) performance status, histological type of cancer, date and cause of death, and the clinical and/or pathologic staging of LC were collected for all patients. Smoking history was categorized into three groups (never, former [a person who had smoked at least 100 cigarettes or cigars during their lifetime and who had quit smoking at the time of the interview], and current-smoker); cumulative smoking amount was calculated in pack-years. Overall survival time was calculated from the date of LC diagnosis to the date of death or last follow-up. Pulmonary function tests were performed at the time of IPF diagnosis.

IPF was diagnosed using the criteria for the usual interstitial pneumonia (UIP) pattern as described by the ATS/ERS/JRS/ALAT 17: sub-pleural, basal, predominantly reticular abnormality or honeycombing, with or without traction bronchiectasis, and the presence of a consistent UIP pattern. Detailed histories were obtained regarding patients' IPF and serologic tests performed to exclude connective tissue disease. A chest computed tomography (CT) scan showing a definite UIP pattern was considered confirmatory of IPF. When the CT scans were not definitive for the presence or absence of IPF, these diagnoses were confirmed at each hospital by a team of specialists in pulmonary medicine, radiology, and pathology.

Categorical variables were compared using Pearson's chi-square test and continuous variables using independent sample t-test to compare baseline characteristics of GAP I and GAP II/III patients. Results for continuous variables are reported as mean with standard deviation, while categorical variables are reported as numbers and percentages. We further investigated the relationships between clinical parameters and mortality using Cox proportional hazard models with stepwise selection of variables found to be significant in the univariate regression analysis. As age, gender, FVC, and DLco are included in the GAP index, they were excluded from the proportional regression and hazard models. A p-value less than 0.05 was used to indicate statistical significance. All statistical analyses were performed using IBM SPSS Statistics (version 25.0).

This research protocol was approved by the Institutional Review Board of Severance Hospital, South Korea (IRB No. 4-2018-0770) and the Institutional Review Board and Ethics Committee of Seoul National University Bundang Hospital (IRB No. B-1707/411-402). The study design was approved by the appropriate ethics review boards, and the requirement to obtain informed patient consent was waived.

## Results

### Baseline characteristics

Baseline characteristics of the study subjects are shown in Table 1. Patients had a median age of 71.0±7.7 years, all but one were men (98.4%), and 54 (88.5%) were either past or current smokers. The cumulative smoking amount (pack-years) was higher among patients with an advanced GAP stage. Hypertension and diabetes mellitus were the most common comorbidities in the early GAP stage group, but tuberculosis and chronic obstructive lung disease (COPD) were diagnosed more frequently in the advanced group.

Table 2 shows the patients' specific characteristics of LC and IPF. There was no significant difference between the GAP groups regarding the diagnostic interval between IPF and LC diagnoses (1445.4 days vs. 1281.1 days). Adenocarcinoma was the most common histology in GAP stage I patients; however, squamous cell carcinoma was more common in stage II/III patients. The most frequent histologic types in early-stage LC patients were squamous cell carcinoma (39.1%) and adenocarcinoma (35.4%); however, adenocarcinoma (32.1%), was more common than squamous cell carcinoma (25.0%) in advanced-stage LC patients.

### Relationship between lung cancer stage and GAP stage

Table 3 shows the univariate regression results for associations between advanced LC stage and clinical factors. A higher LC stage had a significant negative association with BMI (odds ratio [OR]=0.793, 95% confidence interval [CI]=0.649-0.971, *p*=0.025), but a significant positive association with higher GAP stage (OR =5.186, 95% CI=1.589-16.920, *p*=0.006). Age, smoking status, predicted FVC, predicted DLco, and IPF treatment did not significantly affect LC stage.

**Figure 2:**
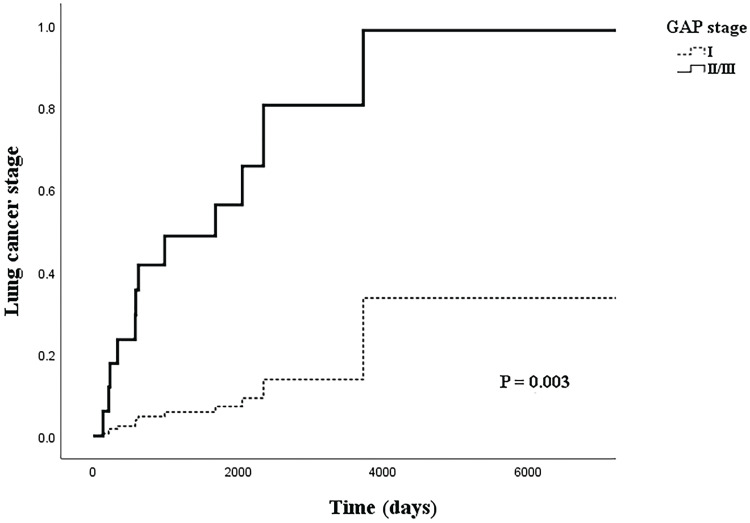
Cox regression model of lung cancer stage by GAP stage, adjusted for idiopathic pulmonary fibrosis treatment and histology.

Table 4 shows the relationship between LC stage and clinical factors in the multivariate logistic regression model. In the logistic regression model, the adjusted OR (aOR) for BMI with LC stage had weakened slightly (aOR=0.710, 95% CI: 0.508-0.992, *p*=0.045). However, the OR for the higher GAP stage group more than doubled from that of the univariate model (aOR=12.158, 95% CI: 1.868-79.138, *p*=0.009). Figure 2 shows the cox regression analysis of LC stage by GAP stage; a similar result was achieved from the proportional hazard model when adjusted for cancer histology and IPF treatment (aOR=11.121, 95% CI: 2.311-53.532, *p*=0.003).

### Survival outcomes

**Figure 3:**
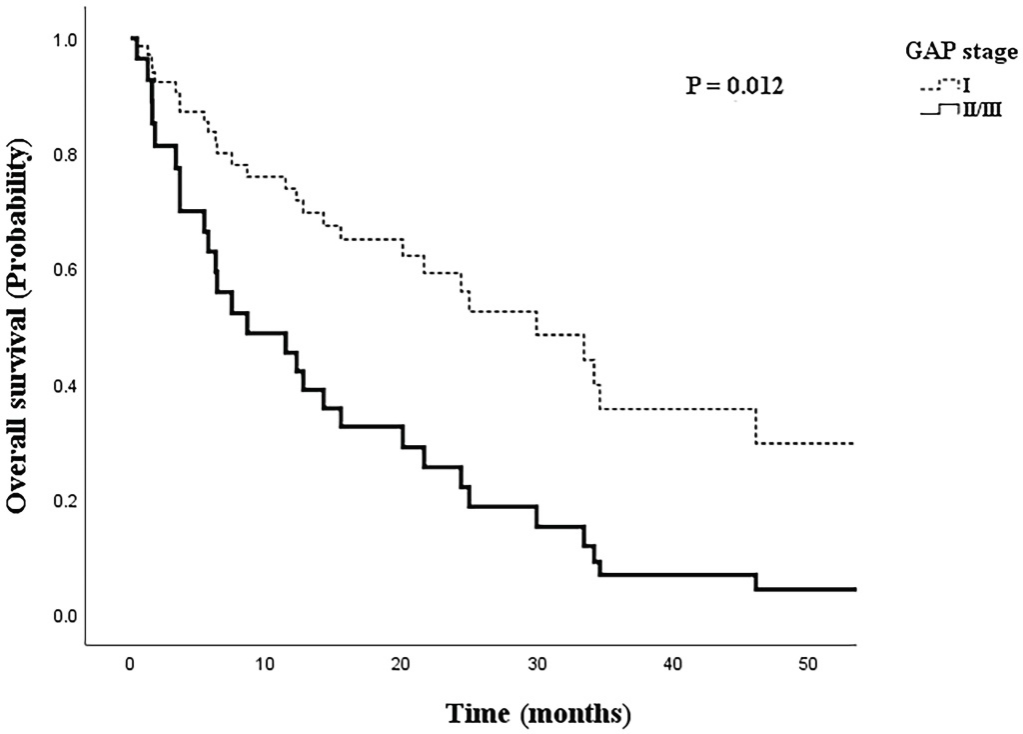
Cox regression model of survival probability by GAP stage, adjusted for IPF treatment, smoking status, body mass index, and histology.

Overall mortality was 58.1% in the early GAP stage group and 83.3% in the advanced GAP stage group, and the mean overall survival time was 20.8±18.3 months in the early GAP stage group and 11.1±12.6 months in the advanced GAP stage group. Figure 3 shows the Cox regression analysis of survival probability by GAP stage, with adjustments for IPF treatment, smoking status, BMI, and histology. There was a significantly lower survival rate in the advanced GAP stage group (aOR=2.860, 95% CI: 1.257-6.508, *p*=0.012).

**Figure 4:**
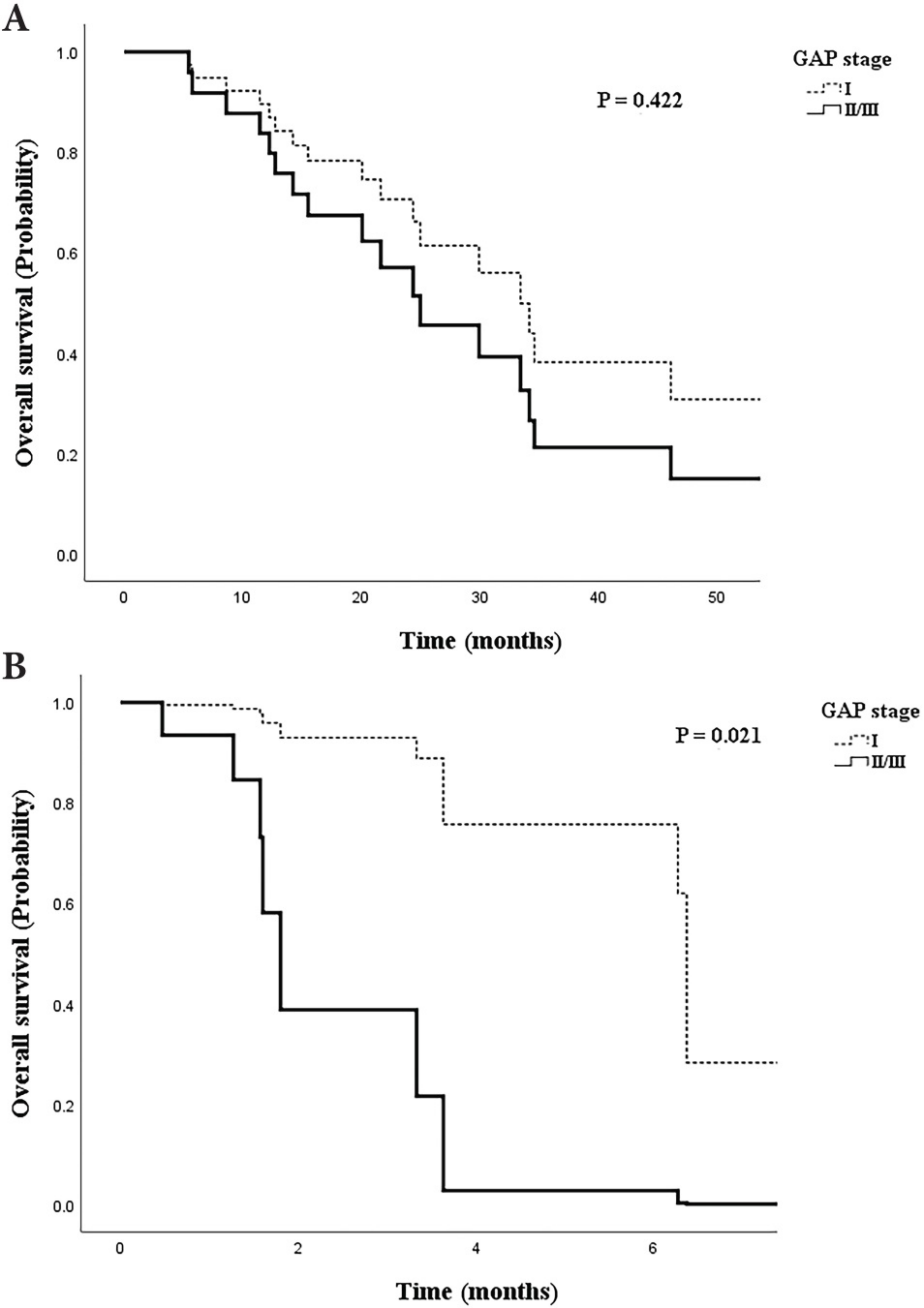
Cox regression model of survival probability adjusted for IPF treatment, smoking status, body mass index, and histology. **A.** Patients with early stage lung cancer by gender-age-physiology (GAP) stage. **B.** Patients with advanced stage lung cancer by GAP stage.

Among patients with early stage LC, overall survival was higher for those in the GAP stage I group after adjustment for cancer histology, BMI, smoking status, and IPF treatment, although the difference was not significant (Figure 4A, aOR=1.613, 95% CI: 0.502-5.184, *p*=0.422). However, the survival of patients with advanced stage LC was significantly higher for those in GAP stage I than in the advanced GAP stages (Figure 4B, aOR=10.412, 95% CI: 1.421-76.285, *p*=0.021).

Table 5 shows the causes of death in the GAP groups. Cancer progression was the main cause of death in the advanced GAP group (38.9%); however, pneumonia (16.3%) and AE-IPF (16.3%) were also dominant in the early GAP stage group.

## Discussion

IPF and LC have common risk factors, and patients with both conditions are known to have a worse prognosis than patients with either condition. This study involved patients with LC that developed after the diagnosis of IPF. Our results showed that IPF severity is associated with the LC stage, but not the interval between IPF and LC diagnoses.

The demographic and clinical characteristics of the patients in this study are similar to those reported in previous studies. Most patients were older men with tobacco exposure 11, 19. Although Ozawa *et al*. 11 did not find differences in survival rates between IPF patients with and without LC, recent studies have reported worse survival outcomes among IPF-LC patients 9, 19, 20. Tomassetti *et al*. 9 reported that the development of LC in IPF patients significantly decreased their median overall survival (IPF with LC: 38.7 months, IPF without LC: 63.9 months; hazard ratio = 5.0; 95% CI: 2.91-8.57; *p* < 0.001). Tzouvelekis *et al.* 21 reported the median survival of IPF-LC patients to be 27.4 and 14.3 months from the time of IPF and LC diagnoses, respectively.

In this report, we have described the differences in clinical features and stages of LC between patients with early and advanced GAP stages. A significant result of our study was the ability to establish an association between IPF severity and LC stage, showing that GAP stage had an independent, positive association with LC stage. This suggests that patients with an advanced GAP stage require more frequent CT scans than the general population in addition to close observation at their routine follow-ups. Even if the LC stage differed according to the GAP stage, there was no significant difference in the time interval between IPF and LC diagnoses (904 days in GAP stage I vs. 959 days in GAP stage II/III).

These two diseases share biological signaling pathways and microenvironments that have been shown to disrupt tissue architecture and lead to dysfunction, contributing to carcinogenesis and fibrosis (e.g., transforming growth factor beta, platelet-derived growth factor, vascular endothelial growth factor, and fibroblast growth factor) 22. Considering the theory that LC developing next to or within fibrosis can cause cellular metaplasia and the longstanding hypothesis that the pathogenesis of tissue damage and abnormal repair are common processes in both IPF and LC, it is reasonable that patients with IPF are vulnerable to the development of cancer 19, 23. Generally, the fibrotic area is broader in patients with an advanced GAP stage than in those with an early GAP stage; therefore, the malignancy would be aroused concomitantly in the widespread areas and result in higher stages of LC.

In patients with early stage LC, the overall survival was higher (though not significantly) in patients with GAP stage I than in those with GAP stage II/III (Figure 4A). However, survival in patients with advanced LC stage differed significantly according to the GAP stage (Figure 4B). This suggests that when LC is at an advanced stage, the disease course could be affected by the GAP stage, which itself is determined by the severity of fibrosis, residual pulmonary functions, and age. A previous study of retrospective data suggested a beneficial effect of preoperative pirfenidone on the incidence of postoperative acute exacerbations in patients with adenocarcinoma and IPF 24. A recent study also suggested that antifibrotic agents should not be discontinued during the diagnostic or therapeutic work-up of LC patients, as benefits seem to outweigh the risk of unfavorable outcomes 1. Our data also suggest that IPF severity affects survival and that clinicians should consider maintaining IPF and anti-cancer treatments, even when the cancer is at an advanced stage. A consensus statement regarding the treatment of these patients needs to be developed.

Conventional chemotherapy and radiotherapy can have deleterious effects on patients with IPF 6, 25, 26, and optimal treatment regimens have not been established. Considering the high incidence and difficulties encountered in treating patients with IPF-LC, those with more severe pulmonary fibrosis are more difficult to treat because of the higher risk of acute exacerbation. The high incidence of LC and its impact on patient survival underscores the importance of clinicians recognizing the predictive factors of LC development and the need for thorough surveillance protocols.

Although our results showed that fibrosis severity may affect LC stage, there was no clinical effect of IPF treatment for delaying LC development (OR 0.889, p=0.867, 95% CI=0.223-3.542). This may have been a reason behind the small number of patients undergoing IPF treatment (n= 8 vs. 7 in both groups, respectively). A cohort study with a larger sample size is needed.

The main strength of our study is that we highlighted the relationship between IPF severity and LC severity, which has rarely been studied.

We recognize that this study did have several limitations: in particular, the small sample size and use of retrospective data. However, we collected the data from two tertiary care hospitals to improve their generalizability. Second, due to the study's retrospective nature, we could not provide a guide for the optimal CT scan interval for LC surveillance in the different GAP groups.

## Conclusion

To our knowledge, this study is the first to show a clear relationship between IPF severity and LC severity. In particular, we found that LC stage is affected by the progression of fibrosis, with higher GAP stages resulting in more advanced cancer stages. These results can provide guidance for treating IPF patients; more frequent checkups and CT scans are warranted to enable early detection of cancer development. Considering the high incidence of LC and its impact on the survival of IPF patients, frequent surveillance and close observation are needed from the time of initial IPF diagnosis. Large and prospective multicenter studies will be key to the development of protocols for the optimal management of patients with IPF-LC.

## Figures and Tables

**Table 1:** Patient baseline characteristics and comorbidities according to GAP stage

Variable	GAP I	GAP II/III	Total	p-value
Total patients, n (%)	43 (70.5)	18 (29.5)	61 (100.0)	
Age (years)	70.6 ± 8.5	72.0 ± 5.6	71.0 ± 7.7	0.524
Sex, male (%)	42 (97.7)	18 (100.0)	60 (98.4)	0.527
BMI, kg/m^2^	23.9 ± 2.4	22.6 ± 4.0	23.5 ± 3.0	0.139
GAP score	2.7 ± 0.6	5.1 ± 1.3	3.4 ± 1.4	<0.001
**Smoking exposure, No. (%)**				0.467
Never	5 (11.6)	2 (11.1)	7 (11.5)	
Former	31 (72.1)	15 (83.3)	46 (75.4)	
Current	7 (16.3)	1 (5.6)	8 (13.1)	
Smoking amount, (Pack-years)	35.6 ± 23.8	47.8 ± 39.8	39.4 ± 29.9	0.152
FVC, % predicted	94.8 ± 15.1	75.0 ± 24.0	86.8 ± 21.3	0.004
DLco, % predicted	74.3 ± 16.7	67.0 ± 20.9	71.0 ± 18.7	0.308
**ECOG**				0.001
0/1/2/3	10/22/6/2	4/6/4/1	14/28/10/3	
**Medical history, n (%)**				
Hypertension	23 (53.5)	7 (38.9)	30 (49.2)	0.298
Diabetes mellitus	14 (32.6)	2 (11.1)	16 (26.2)	0.085
Cardiovascular disease	2 (4.7)	2 (11.1)	4 (6.6)	0.442
Rheumatic disease	2 (4.7)	1 (5.6)	3 (4.9)	0.883
Prior tuberculosis	3 (7.0)	6 (33.3)	9 (14.8)	0.009
COPD	6 (14.0)	5 (27.8)	11 (18.0)	0.204
Other malignancy	9 (21.0)	4 (22.2)	13 (21.3)	0.748
Overall mortality, n (%)	25 (58.1)	15 (83.3)	40 (65.6)	0.140
Median OS, months	20.8 ± 18.3	11.1 ± 12.6	18.2 ± 17.4	0.065

Abbreviations: GAP staging system, gender (G), age (A), forced vital capacity (FVC), and diffusing capacity of carbon monoxide (DLco); BMI, body mass index; COPD, chronic obstructive lung disease.Data are presented as median, interquartile range, or frequency (%).

**Table 2:** Characteristics of LC and IPF according to GAP stage

Variable	GAP I	GAP II/III	Total	p-value
Time from IPF to LC* (day)	1445.4 ±1420.1	1281.1±983.4	1396.9±1300.5	0.662
The number of CT scans§	2.63 (2.0, 19.0)	2.41 (2.0,7.0)	2.56 (2.0,19.0)	0.714
Period from IPF to LC/CT count (day)	626.1± 599.5	581.2±484.4	612.9±564.4	0.780
**Cancer treatment, n (%)**				
Operation	23 (54.5)	3 (16.7)	26 (42.6)	
Cytotoxic chemotherapy	22 (51.2)	3 (16.7)	25 (41.0)	
Targeted/Immunotherapy	6 (14.0)	1 (5.6)	7 (11.5)	
CCRT	11 (25.6)	0 (0.0)	11 (18.0)	
Radiotherapy alone	5 (11.6)	3 (16.7)	8 (13.1)	
Conservative care	2 (4.7)	5 (27.8)	7 (11.5)	
**Clinical lung cancer stage, n (%)**				0.005
I to IIIA (early)	33 (82.5)	7 (17.5)	40 (65.6)	
IIIB to IV (advanced)	10 (47.6)	11 (52.4)	21 (34.4)	
IPF treatment, n (%)	8 (18.6)	7 (38.9)	15 (24.6)	0.162
**Histologic type, n (%)**				0.063
Small cell carcinoma	10 (23.3)	4 (22.2)	14 (23.0)	
Adenocarcinoma	18 (41.9)	4 (22.2)	22 (36.1)	
Squamous cell carcinoma	14 (32.6)	9 (50.0)	23 (37.7)	
Other	1 (2.3)	1 (5.6)	2 (3.3)	

Abbreviations: GAP staging system, gender (G), age (A), forced vital capacity (FVC), and diffusing capacity of carbon monoxide (DLco); CT, computed tomography; CCRT, concurrent chemoradiotherapy; ECOG, Eastern Cooperative Oncology Group; RT, radiotherapy; CTx, chemotherapy; OS, overall survival.Data are presented as median, interquartile range, or frequency (%). *Time from IPF to LC, time gap between diagnosis of lung cancer and diagnosis of IPF (days). ^§^The number of CT scan was described with mean (minimum, maximum value).

**Table 3:** Results from the univariate logistic regression analysis of clinical factors associated with advanced lung cancer stage

Variable	OR	95% CI	p-value
Age (years)	1.037	0.965-1.115	0.317
BMI, kg/m^2^	0.793	0.649-0.971	0.025
Ever smoker	0.814	0.125-5.306	0.830
FVC % predicted	0.984	0.961-1.009	0.211
DLco % predicted	0.999	0.977-1.021	0.929
GAP stage (Stage I vs. II/III)	5.186	1.589-16.920	0.006
IPF treatment	0.889	0.223-3.542	0.867
Time Gap between diagnosis of lung cancer and diagnosis of IPF, days	1.000	1.000-1.001	0.514

Abbreviations: GAP stage system, gender (G), age (A), forced vital capacity (FVC), and diffusing capacity of carbon monoxide (DLco); BMI, body mass index; OR, odds ratio; CI, confidence interval.

**Table 4:** Results from the multivariate logistic regression analysis of clinical factors associated with early vs. advanced lung cancer stage

Variable	OR	95% CI	p-value
BMI, kg/m^2^	0.710	0.508-0.992	0.045
Ever smoker	0.787	0.091-6.799	0.828
GAP stage (Stage I vs. II/III)	12.158	1.868-79.138	0.009
IPF treatment	0.558	0.078-4.010	0.562

Abbreviations: GAP stage system, gender (G), age (A), forced vital capacity (FVC), and diffusing capacity of carbon monoxide (DLco); BMI, body mass index; OR, odds ratio; CI, confidence interval.

**Table 5:** Comparison of the causes of death according to GAP stage

Cause of death, n (%)	GAP I (n=43)	GAP II/III (n=18)	Total (n=61)
Pneumonia	7 (16.3)	3 (16.7)	10 (16.4)
RT/CTx pneumonitis	2 (4.7)	1 (5.6)	3 (4.9)
Cancer progression	7 (16.3)	7 (38.9)	14 (23.0)
AE-IPF	7 (16.3)	3 (16.7)	10 (16.4)
*Other causes	2 (4.7)	1 (5.6)	3 (4.9)
Total	25 (58.1)	15 (83.3)	40 (65.6)

*Other causes: Subdural hemorrhage and chronic renal failure in GAP I group; systemic viral infection in GAP II/III group. Abbreviations: RT, radiotherapy; CTx, chemotherapy; AE-IPF, acute exacerbation of idiopathic pulmonary fibrosis.

## Abbreviations

FVC: forced vital capacity
GAP: gender-age-physiology
DLco: diffusing capacity of the lung for carbon monoxide
BMI: body mass index
ECOG: Eastern Cooperative Oncology Group
COPD: chronic obstructive lung disease
OS: overall survival
IPF: idiopathic pulmonary fibrosis
LC: lung cancer
CT: computed tomography
CEA: carcinoembryonic antigen
IQR: interquartile range
CI: confidence interval
